# Paralyzing Action from a Distance in an Arboreal African Ant Species

**DOI:** 10.1371/journal.pone.0028571

**Published:** 2011-12-14

**Authors:** Aline Rifflet, Nathan Tene, Jerome Orivel, Michel Treilhou, Alain Dejean, Angelique Vetillard

**Affiliations:** 1 Venoms and Biological Activities Laboratory, EA 4357, PRES-Université de Toulouse, Jean-François Champollion University Center, Albi, France; 2 Centre National de la Recherche Scientifique (UMR-8172), Ecologie des forêts de Guyane, Campus Agronomique, Kourou, France; 3 Université de Toulouse, Université Paul Sabatier, Toulouse, France; University of Arizona, United States of America

## Abstract

Due to their prowess in interspecific competition and ability to catch a wide range of arthropod prey (mostly termites with which they are engaged in an evolutionary arms race), ants are recognized as a good model for studying the chemicals involved in defensive and predatory behaviors. Ants' wide diversity of nesting habits and relationships with plants and prey types implies that these chemicals are also very diverse. Using the African myrmicine ant *Crematogaster striatula* as our focal species, we adopted a three-pronged research approach. We studied the aggressive and predatory behaviors of the ant workers, conducted bioassays on the effect of their Dufour gland contents on termites, and analyzed these contents. (1) The workers defend themselves or eliminate termites by orienting their abdominal tip toward the opponent, stinger protruded. The chemicals emitted, apparently volatile, trigger the recruitment of nestmates situated in the vicinity and act without the stinger having to come into direct contact with the opponent. Whereas alien ants competing with *C. striatula* for sugary food sources are repelled by this behavior and retreat further and further away, termites defend their nest whatever the danger. They face down *C. striatula* workers and end up by rolling onto their backs, their legs batting the air. (2) The bioassays showed that the toxicity of the Dufour gland contents acts in a time-dependent manner, leading to the irreversible paralysis, and, ultimately, death of the termites. (3) Gas chromatography-mass spectrometry analyses showed that the Dufour gland contains a mixture of mono- or polyunsaturated long-chain derivatives, bearing functional groups like oxo-alcohols or oxo-acetates. Electrospray ionization-mass spectrometry showed the presence of a molecule of 1584 Da that might be a large, acetylated alkaloid capable of splitting into smaller molecules that could be responsible for the final degree of venom toxicity.

## Introduction

Ants dominate the invertebrate communities in tropical rainforest canopies, often representing ca. 50% of the animal biomass and 90% of the individuals. This is possible because most species are partially herbivorous, feeding on pollen, extrafloral nectar and food bodies, or are considered “cryptic herbivores” when they attend sap-sucking hemipterans for their honeydew [1]–[3]. “Territorially-dominant” arboreal ants, whose very populous colonies defend absolute territories (several neighboring trees) distributed in a mosaic pattern creating what has become known as “arboreal ant mosaics” [4]–[7], account for much of this abundance.

Territorially-dominant arboreal ants are characterized by their intra- as well as interspecific territoriality, extremely populous colonies, the ability to build large and/or polydomous nests (but certain species can nest in hollow and/or dead branches), their ability to attend hemipterans to obtain the sugar-rich honeydew that fuels their energy-costly territoriality, and their skill at capturing a wide range of prey [4]–[7]. Since most prey in the tree foliage are insects able to escape by flying away, jumping or dropping, arboreal ants have optimized their ability to capture such insects in this restricted foraging area. In the territorially-dominant arboreal ant species studied so far, workers ambush in a group permitting them to capture a wide range of insects that are spread-eagled, and only certain species need to use their venom (or pygidial gland secretion for Dolichoderinae) [7]–[11]. Note that spread-eagling the prey is possible based on the ability of workers discovering a prey to recruit nestmates situated within a radius of 20–30 cm thanks to the emission of a pheromone. This ‘short-range recruitment’ can be distinguished from ‘long-range recruitment’ that occurs when a foraging worker, not necessarily the individual discovering a large food source (or a large prey) returns to its nest laying a scent trail to recruit nestmates [12]. In a non-dominant arboreal species, the workers, which hunt solitarily, capture a wide range of prey using their venom; however, when confronted with termites or competing arboreal ants defending a sugary food source, they use volatile secretions produced by their mandibular gland. The action at a distance of these secretions keeps them from having to come into contact with dangerous enemies [13].

Large colonies of some ground-nesting, plant-foraging ant species can defend their territories from territorially-dominant arboreal ants. This is the case in Central and West Africa for *Crematogaster striatula* in cocoa tree plantations with low canopies and along forest edges [4], [5], [14]. Also, during the formation of forests, as the trees grow and age, there is a succession of different associated ants. In this type of situation, the chemical defenses of the workers permit the *C. striatula* colonies to delay colonization by territorially-dominant arboreal ants that are the last to arrive [15]. The *C. striatula* colonies nest in the soil, in rotten branches lying on the ground and in termitaries [16], [17]; however, during studies on ant diversity [18], a relatively high number of foraging workers were gathered from the ground. They do indeed forage on the ground where they prey mostly on termites (AD, pers. obs.) even though termites have developed elaborate architectural, behavioral, morphological and chemical means of defense against ants [19].

The way in which the gaster is attached to the thorax in the genus *Crematogaster* means that it can be bent forwards over the alitrunk so that the abdominal tip can be pointed in nearly any direction. In most species the venom is frothy, accumulates on the tip of the spatulate stinger – not a suitable device for injecting venom – and is spread on the tegument of prey and enemies on which it acts topically [20]–[21]. Air-borne recruitment pheromones are also emitted by the protruded stinger [20], [22]–[25]. Indeed, two glands are connected to the stinging apparatus of aculeate hymenopterans: the poison or acid gland and the Dufour gland corresponding to the colleterial gland, also known as the alkaline gland [26]–[31]. In the genus *Crematogaster*, the hypertrophied Dufour gland is considered to be the point of origin of the venom [24], [27], [32]. Chemical analyses of Dufour gland secretions have been conducted on several *Crematogaster* species from Europe [22], [33], [34], Papua New Guinea [35], Brazil [36]–[38], Georgia [39] and Kenya [40]. These analyses have shown the secretions to be a valuable source of active defensive compounds, including long-chain conjugated dienones, furan, and trihydroxylated cyclohexane derivatives, as well as diterpenes. In three European *Crematogaster* species, the compounds from the Dufour gland mix with enzymes stored in the poison gland, resulting in the formation of highly toxic aldehydes during the emission of venom [22], [34], [41].

We hypothesized that the secretions of the workers – used both defensively and offensively – are particularly potent. To date, toxicity tests have been conducted on only five *Crematogaster* species and have shown the repellent activity of the venoms of *C. scutellaris*, *C.* sp. prox. *abstinens, C. distans* and *C. brevispinosa rochai* on other ants [21], [22], [36], [41]. Due to the thin cuticle on their abdomen, however, termites are much more sensitive to this venom than ants [42], [43]. We consequently adopted a three-pronged research approach. (1) We studied the termite capture behavior of *C. striatula* workers as well as their reactions when confronted with workers of another ant species, (2) we conducted bioassays to evaluate the toxicity of the Dufour gland contents, and (3) we analyzed these contents.

## Materials and Methods

All of our observational and field studies were conducted according to European laws for scientific research currently in vigor. No permits were required for the collection of *Reticulitermes grassei* workers from Albi (France) or for *C. striatula* from Yaoundé, Cameroon and its vicinity. Indeed, these species are not protected.

### Predatory behavior and confrontations with competing ant species

These studies were conducted in the field on four colonies. In each case, we first installed plastic trays (80×40 cm) on the ground 60 cm from the nest entrances. During 1 week, we allowed the workers to mark these trays that we then used as experimental territories.

We compared the predatory behavior of the ant workers when confronted with termite workers (*Macrotermes bellicosus*; Macrotermitidae; 35 cases) (5–7.2 mm long; 10±0.4 mg; N = 30) and large termite soldiers (20 cases; 13 mm long; 21±0.3 mg; N = 20). These prey were larger than the *Crematogaster* workers (3.0- to 3.5-mm total length; 1.2±0.1 mg; N = 30). The day of the tests, we placed prey one at a time on the trays, and we observed and recorded the behavioral sequences from the introduction of the prey into the center of the hunting areas (on the plastic trays) until they were captured and retrieved to the nest (see an example in Richard et al. [8]). The different phases of the behavioral sequence were: detection, alarm posture, short-range recruitment, venom emission (the prey then falls down) and prey retrieval. Two successive observational periods were separated by at least 30 minutes.

After the *C. striatula* colonies became accustomed to hunting on the trays and we had completed the study of their predatory behavior, we instigated the workers from neighboring *Camponotus brutus* (Formicinae) colonies to forage on the trays (media workers: 12–13 mm; 8–9 mg). For this, we deposited very small drops of honey between their nests, and, when no *C. striatula* were present, we deposited a larger drop of honey on the trays. After ca. 20 minutes, the *C. brutus* worker that first discovered the large drop of honey recruited nestmates. In a few minutes, 4 to 12 *C. brutus* workers imbibed the honey until a *C. striatula* individual beginning to forage discovered both their presence and that of the honey. We noted the reactions of the opponents during these encounters (24 cases). We were able to conduct similar experiments with one colony of *Oecophylla longinoda* (Formicinae) and another colony of *Tetramorium aculeatum* (Myrmicinae) (10 cases for each colony). Indeed, from time to time workers from these two territorially-dominant arboreal ant species forage on the ground [7].

For statistical comparisons (unpaired Student's *t*-test), we used GraphPad Prism 5.0 software.

### Bioassays

Three colonies with queens, workers and brood were collected from Yaoundé, Cameroon and its vicinity and placed into plastic containers (35 cm×25 cm×15 cm) whose walls were coated with antistatic silicone spray to prevent the ants from climbing out. Then, before the containers were sealed for transportation to the laboratory in France, we placed two plastic tubes containing pieces of cotton soaked in water and honey, respectively, inside them. Once in the laboratory, these containers were provided with a watering place and connected to a foraging area (another plastic box) and kept at ca. 85% relative humidity. The ants were regularly provided with a honey/water mixture and mealworms placed in the foraging area.

Because *C. striatula* workers commonly prey upon termites, the toxicity of their venom was examined using *Reticulitermes grassei* (*Rhinotermitidae*) workers collected from Albi, France (average body weight: 2.9±0.1 mg; N = 30).

Dissected Dufour glands (N = 200) were extracted in 250 µL hexane (chromatography grade, Merck), macerated and centrifuged at 1500 g at room temperature for 2 min. The supernatant was collected. The contents of 100 glands were lyophilized and weighed, resulting in 45 µg for one *C. striatula* Dufour gland. Otherwise, the collected supernatant was evaporated and dissolved in pure dichloromethane (range of dilutions: 0, 90, 270 and 540 µg/µL of Dufour gland extract), using a method adapted from Marlier et al. [21], since the liquid obtained from the Dufour gland is not soluble. The glands are kept at −20°C until the experiments could be conducted.

A 5 µL Hamilton syringe calibrated into 0.5 µL divisions was used to deposit 0.5 µL of Dufour gland extracts (at different dilutions) on the abdomens of the tested termites that were held carefully with a pair of smooth forceps. Then, the termites were kept in darkness in a Petri dish at room temperature (ca. 22°C) and observed 5 minutes, ½ hour, and 1, 2, 4, 6, 8, 12 and 24 hours after contact with the extract. For each dilution, the experiments were carried out using 90 termites. All of the tests were conducted at room temperature (ca. 22°C) to simulate natural conditions. Behavioral classifications were adapted from Boevé [44] with the following parameters: normal (N: walking and moving the body normally, mandibles responsive to stimulation, continuous movement of the antennae), paralysis (P: incapacity to walk or move the body normally, sometimes accompanied by uncoordinated contractions of the legs, although the mandibles and antennae remain responsive) or dead (D: total absence of movement, including mandibles and antennae that were not responsive; in this case, the termites that had rolled onto their backs did not move their legs and did not react when we touched them with the forceps or pinched their appendices).

We used the Friedman test and a *post-hoc* test to compare the effect of time and treatments of the *Crematogaster striatula* Dufour gland extracts on termite workers (R software, see [45]–[47] for the *post-hoc* comparisons using R software).

### Dufour gland extracts chemical profile

A gas chromatography-mass spectrometry analysis (GC-MS) was carried out on the contents of three Dufour glands using a Trace GC 2000 coupled with an ion trap mass spectrometer (Polaris Q; Thermo-Finnigan; Courtaboeuf, France) with Xcalibur-software-based data acquisition and an autosampler (AS 2000). The gas chromatograph was equipped with an RTX5-MS (5% diphenyl: 95% dimethylpolysiloxane) capillary column 30 m×0.25 mm i.d. (0.25 µm film thickness). The injector temperature was 280°C. Samples were injected in the splitless mode. The oven temperature was as follows: an initial temperature of 120°C, increased to 280°C at 5°C min^−1^, and then held for 5 min. The carrier gas was helium (alphagaz 2; Air Liquide; Labège, France) used at a flow rate of 1.0 mL.min^−1^. A 1 µL volume was injected splitless, with the split valve closed for 1 min. The mass spectrometric detector was operated first in electron impact (EI) ionization mode with an ionizing energy of 70 eV; the ion source and transfer line temperatures were 230°C and 220°C, respectively. When used, chemical ionization (CI) had an ionizing energy of 120 eV; the ion source and transfer line temperatures were 180°C and 190°C, respectively. In both cases, acquisition begins after 4 minutes and scanning from m/z 60 to 500.

Also, 30 Dufour glands were preserved in hexane, dried, diluted in 200 µL of methanol then introduced into the mass spectrometer working in positive mode electrospray ionization (+ESI-MS) at a flow rate of 5 µL.min^−1^. The syringe was connected to the ESI probe with a fused silica capillary. The ion trap mass spectrometer (LCQ Advantage; Thermo-Finnigan; Courtaboeuf, France) with Xcalibur-software-based data acquisition was operated under the following conditions: 300°C (capillary temperature); 50 and 5 psi (sheath gas and auxiliary gas, respectively); 4 kV (capillary voltage); 150–2000 (scan range). For the acquisition of MS-MS, the automated data dependent acquisition functionality was used. The analysis was conducted twice with two lots of 30 glands extracted by two different researchers.

## Results

### Predatory behavior and confrontations with competing ant species

**Figure 1:**
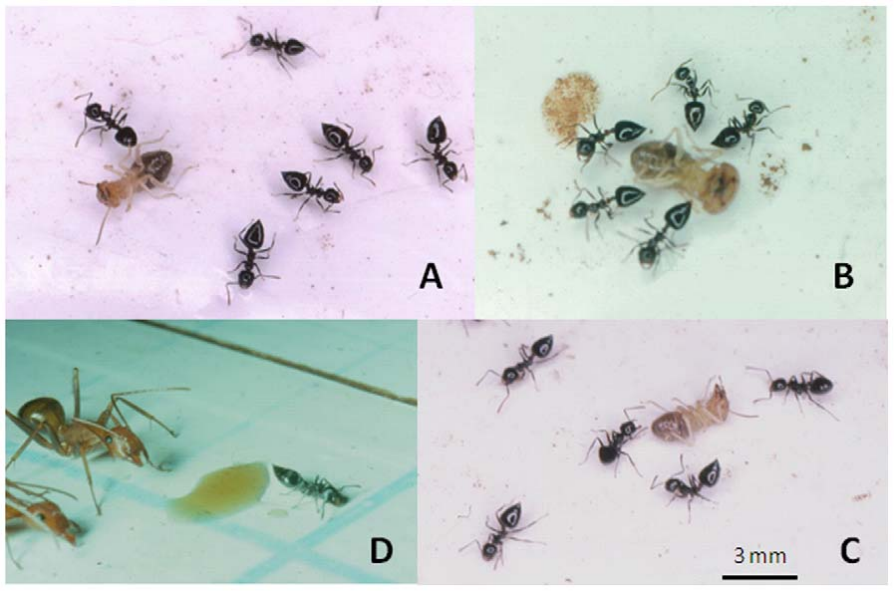
*Crematogaster striatula* ants capturing a termite worker. Workers firstly surrounded the termite at a distance of 5–10 mm, their abdominal tip pointed toward the prey. **A.** After ca. 10 minutes the termite fell down and rolled onto its back, its legs batting the air. One ant approached it very slowly. **B.** When there were fewer movements of its legs, all of the ants approached the termite. **C.** Later the ants prepared to seize the termite by an appendage to retrieve it to their nest. **D.** Interspecific competition. A *Crematogaster striatula* worker that had discovered several *Camponotus brutus* imbibing honey on its territory caused them to retreat by very slowly approaching in a backward movement, with its abdominal tip pointed toward the aliens. No contact between the antagonists was noted.

The predatory behavior of *C. striatula* workers was stereotypical as we did not note any fundamental differences in their reactions based on the different prey tested. While several *C. striatula* workers were foraging on the trays, one of them detected the presence of the prey at a very short distance (3–6 mm) and immediately took up an alarm posture typical of *Crematogaster* ants with the gaster held high and the stinger protruded. This resulted in the recruitment at short range of nestmates foraging in the vicinity (ca. 15 cm). Each arriving individual pivoted when 6–10 mm from the prey, and oriented its abdominal tip toward it. In less than 30 seconds, up to 15 workers surrounded the prey at a distance of 5–10 mm, all with their protruded stinger oriented toward the prey (Fig. 1A–C). They then approached it by backing up until their stinger was very close to the prey (Fig. 1B). We never noted the formation of froth at the extremity of the stinger as for certain other arboreal *Crematogaster* species. In the 10 minutes following their detection, the termites began to shake. Then, they fell down and rolled onto their backs, their legs batting the air (Fig. 1A–C). The workers approached them very slowly, but did not touch them, or only by accident, until the termites' movements became sluggish. At this time, one or as many as four workers seized the termites and retrieved them to the nest.

The slight differences we noted according to prey type refer to the number of *C. striatula* workers recruited or involved in retrieving the prey. Up to six ants, their stinger extruded, surrounded termite workers, while eight to 15 ants surrounded termite soldiers (5.11±0.1 *vs*. 11.85±0.48 ants; dl = 53; Student's *t*-test = 17.7; P<0.0001). Termite workers were retrieved by one to three *C. striatula* ants, while termite soldiers were retrieved by three to five ants (1.6±0.1 *vs*. 4.05±0.15 ants; dl = 53; *t* = 13.8; P<0.0001). Nevertheless, the time separating the discovery of the prey from the moment when a worker began to seize one of its appendages to retrieve it was not significant (24.8±0.73 *vs*. 24.25±0.87 minutes; dl = 53; *t* = 0.46; P = 0.64). This is likely due to the fact that the number of workers involved in mastering the prey was adjusted to each situation (ca. twice as many *C. striatula* ants were involved in mastering termite soldiers that weigh ca. twice as much as termite workers).

Each *C. striatula* worker discovering a group of *Camponotus* imbibing honey on its territory immediately protruded its stinger when situated 6–8 mm from the closest *Camponotus*. Then, it pivoted, orienting its protruded stinger toward the group of aliens. While the *C. striatula* worker moved backward very slowly, the *Camponotus* workers, even if numerous (up to 12 individuals), abandoned the drop of honey one after the other without showing any aggressive behavior. They abandoned the drop of honey in all 24 cases tested; no contact was noted between the opponents (Fig. 1D). Meanwhile, some *C. striatula* workers were recruited at short range and participated in the exclusion of the *Camponotus* by orienting their protruded stinger toward these aliens. After the *Camponotus* were excluded, the *C. striatula* workers surrounded the drop of honey to imbibe it. The same was noted during the confrontations with *Oecophylla* and *Tetramorium* workers (10 confrontations in each case).

### Bioassays

To have a control lot, we placed 90 µg of pure dichloromethane on termite workers; 96.7% of the individuals remained active after 24 hours (N = 90; Table 1). In the experimental lots where we deposited 0.5 µL of Dufour gland extracts at different dilutions, the paralysis and death of the termites occurred in a dose- and time-dependent manner (Table 1). For all of the concentrations tested, while the “normal state” of the termites regularly declined, the incidence of paralysis and then death increased. For a concentration of 270 µg/µL of extracts (corresponding to three Dufour glands), for example, paralysis, noticeable after 5 minutes, affected 16.7% of the individuals in 30 minutes and regularly increased, and the incidence of death of the termites particularly increased after 6 hours. In 24 hours, all of the tested termites were dead (Table 1).

**Table 1:** Comparison of the effect of time and doses of the *Crematogaster striatula* Dufour gland extracts applied topically on termite workers.

	5 min	30 min	1 h	2 h	4 h	6 h	8 h	12 h	24 h
Control	N: 90	N: 89	N: 89	N: 89	N: 88	N: 87	N: 87	N: 87	N: 87
	P: —	P: 1	P: 1	P: —	P: 1	P: 2	P: 1	P: —	P: —
	D: —	D: —	D: —	D: 1	D: 1	D: 1	D: 2	D: 3	D: 3
1 gland	N: 85	N: 82	N: 79	N: 72	N: 58	N: 33	N: 27	N: 17	N: —
90 µg/µL	P: 4	P: 7	P: 7	P: 14	P: 28	P: 33	P: 25	P: 14	P: 2
	D: 1	D: 1	D: 4	D: 4	D: 4	D: 24	D: 38	D: 59	D: 88
3 glands	N: 81	N: 78	N: 69	N: 63	N: 51	N: 36	N: 12	N: 3	N: —
270 µg/µL	P: 6	P: 9	P: 18	P: 21	P: 33	P: 39	P: 6	P: 3	P: —
	D: 3	D: 3	D: 3	D: 6	D: 6	D: 15	D: 72	D: 84	D: 90
6 glands	N: 75	N: 66	N: 58	N: 50	N: 45	N: 37	N: 21	N: 4	N: —
540 µg/µL	P: 14	P: 20	P: 28	P: 35	P: 40	P: 33	P: 22	P: 10	P: —
	D: 1	D: 4	D: 4	D: 5	D: 5	D: 20	D: 47	D: 76	D: 90

The doses correspond to the equivalent of one, three or six Dufour gland extracts (N: normal state of the termites that are active; P = paralysis; D: Death). Statistical comparison. Friedman χ^2^ = 20.8; df = 3; P = 0.00011; *Post-hoc* tests; control *vs*. 1 gland: NS; control *vs*. 3 glands: P = 0.0011; control *vs*. 6 glands: P = 0.0002.

### Composition of the Dufour gland extracts

**Figure 2:**
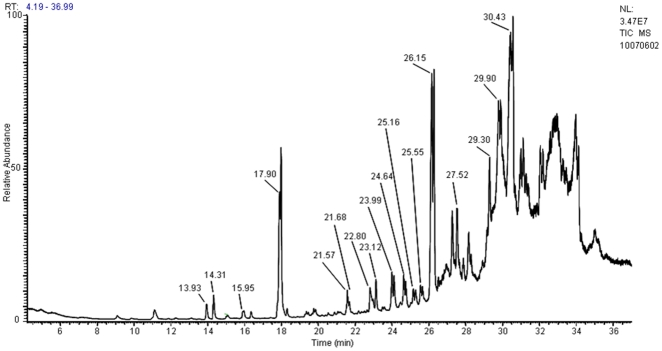
GC-MS TIC traces of the Dufour gland extracts of *Crematogaster striatula* by electronic impact ionization (EI): injection of 1 µL of hexanic extract. The major double peak at a retention time of 26.15 minutes, for example, corresponds to two isomers of compound C21H30O3 (m/z 330). The trace shows other double or single peaks corresponding to C16 at C23 compounds.

The chromatograms of the Dufour gland extracts show the presence of ca. 50 compounds whose mass ranges from 178 to 348 Da (Fig. 2). Among them, we identified two C16, four C18, four C19, twelve C21 and four C23 long-chain derivatives. We recognized, for example, oxo-acetate isomeric compounds with a mass of 330 Da corresponding to the empirical formula C_21_H_30_O_3_ that were also present in the Dufour gland contents of *C. scutellaris* [33], [34].

As shown in Table 2, several components have a similar mass (for example, five compounds have a mass of 330 Dalton, corresponding to the empirical formula C_21_H_30_O_3_). When we compare electronic ionization and chemical ionization mass spectra, they indicate the presence of positional or conformational isomers around the double bonds. The appearance of several double peaks supports this hypothesis. Assuming that we can isolate these components and that we have a sufficient quantity, their structure may be determined through nuclear magnetic resonance as was done for *Crematogaster* spp. [33], [34].

**Table 2:** Mass of the peaks from Fig. 2 and the corresponding identification according to Daloze et al. 1987, 1991 [33], [34].

Retention time (min.)	Compounds	identified ions (m/z)		Mass
		EI	CI	
13,93	C_16_H_30_O	55, 67, 81	239, 267	238
14,31	C_16_H_32_O	55, 67, 81	241, 269	240
15,9	C_18_H_32_O	55, 67, 79, 93, 121, 135, 149	265, 282	264
15,95	C_18_H_32_O	55, 67, 79, 93, 121, 135, 149	265, 282	264
17,9	C_18_H_34_O	55, 67, 81, 121, 135, 149	249, 267, 284	266
17,98	C_18_H_34_O	55, 67, 81, 121, 135, 149	249, 267, 284	266
21,57	C_19_H_40_O	67, 79, 97	261, 279, 296	278
21,68	C_19_H_40_O	67, 79, 97	279	278
22,8	C_21_H_34_O	79, 94, 121, 302	285, 303	302
23,12	C_21_H_36_O	94, 107, 304	305	304
23,99	C_19_H_30_O_3_	67, 79	289, 307, 324	306
24,64	C_19_H_30_O_3_	67, 79	289, 307, 324	306
24,70	C_21_H_30_O_3_	67, 81, 94	331	330
25,55	C_21_H_30_O_3_	67, 79, 94	319, 331	330
25,66	C_21_H_30_O_3_	67, 79, 94	301, 319, 331	330
26,15	C_21_H_30_O_3_	67, 79, 94, 107, 330	331	330
26,29	C_21_H_30_O_3_	67, 79, 94, 107, 330	331	330
27,26	C_21_H_36_O_3_	67, 79, 95, 121	285, 303, 321, 338	320
27,52	C_21_H_38_O_3_	67, 83, 95, 121	323, 340	322
27,88	C_21_H_34_O_2_	67, 77, 91, 105, 133	273, 283, 301, 319, 336	318
28,17	C_21_H_32_O_3_	67, 79, 95, 110, 123	333, 350	332
29,30	C_21_H_32_O_3_	67, 79, 95, 110, 123	315, 333	332
29,90	C_21_H_32_O_3_	67, 79, 95, 110, 123	315, 333	332
30,4	C_23_H_40_O_2_	350, 331, 121, 95, 79, 67	313, 331, 349, 366	348
30,57	C_23_H_40_O_2_	350, 331, 121, 95, 79, 67	313, 331, 349, 366	348
31	C_23_H_40_O_2_	346, 331, 305, 151, 79/81, 67	305, 329, 347, 364	346
31,11	C_23_H_40_O_2_	346, 331, 305, 151, 79/81, 67	305, 329, 347, 364	346

Note that several components have similar masses and fragmentations.

The GC-MS analysis with electronic impact ionization (EI) showed, after fragmentation, the presence of linear mono- and polyunsaturated hydrocarbonated chains with functional groups such as ketone, alcohol, and ester functions (Fig. 2). Chemical ionization (CI) using either ammonia or methane allowed us to identify the molar mass of each peak. Based on the nature of the compounds, we noted two types of adducts: (1) the protonated compound [M+H]^+^, and (2) the compound linked to an ammonium ion [M+18]^+^. Furthermore, the presence of alcohol groups are related to the loss of water molecules [M+H-n18]^+^, with “n” corresponding to the number of alcohol groups present in the compound.

**Figure 3:**
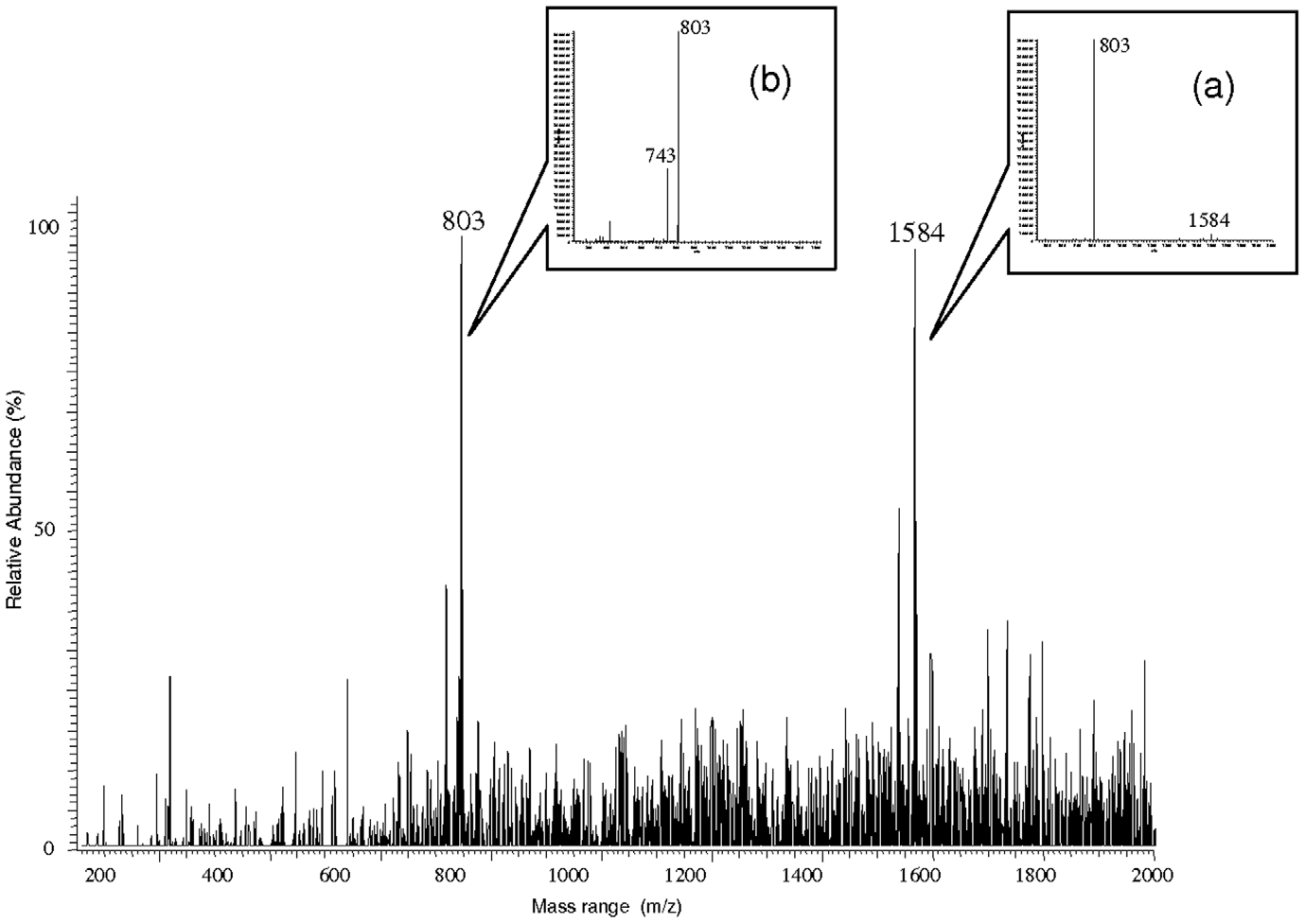
Mass spectra collected as total ion current (TIC) from the direct infusion analysis of Dufour gland extracts from *Crematogaster striatula* (positive mode ESI-MS). We can see two major peaks at m/z 1584 and 803. (a) MS-MS of m/z 1584: the total fragmentation results only in the ion m/z 803, showing that this ion is a part of ion 1584. (b) MS-MS of the ion m/z 803: partial fragmentation with only the loss of 60 u (the loss of an acetyl radical) resulting in the ion m/z 743.

Although the behavior of the *C. striatula* workers indicates that the defensive-offensive products emitted during confrontations is volatile, we investigated further using positive mode ESI-MS to verify if other polar or less volatile compounds also exist and so act as precursors in the Dufour gland extracts (see venom precursors in Pasteels et al. [22]). The spectrum obtained by infusion showed the presence of compounds previously identified using CI-GC-MS (e.g., m/z 331, [C_21_H_30_O_3_+H]^+^), plus two mains peaks with a relatively high mass (m/z 803 and m/z 1584) (Fig. 3). The use of the zoom-scan mode to attain isotopic resolution showed the presence of monocharged ions (M+H)^+^. The MS-MS on ion 803 is consistent with the presence of a fragment of 743 Da (803-60) corresponding to the loss of acetate (Fig. 3b). Furthermore, the MS-MS on m/z 1584 breaks into ion m/z 803 plus another molecule of m/z 781 not visible here due to the technique used (Fig. 3a); the latter might break into fragments in turn. Peaks 1584 and 803 represent, in fact, one acetylated molecule (with ion 803 as a part of it). At this point, it is difficult to further characterize this quite high mass compound. Indeed, attempts at fragmenting ion 803 failed, resulting only in the loss of acetate, which means that this compound is stable as a cyclic structure. The fact that this compound appears at the m/z 1584 due to (M+H)^+^ indicates that its mass is odd (1583 Da), meaning that it contains one (or an odd number) of nitrogen atoms and might be a large, acetylated alkaloid.

## Discussion

table-1-captionWe show in this study that *C. striatula* workers emit chemicals from their protruded stinger that have three functions. First, nestmates situated in the vicinity are attracted through a phenomenon known as “short range recruitment” (as opposed to “long-range recruitment” meaning that a worker returns to its nest to solicit nestmates [12]). Second, a defensive role was noted as alien ants were repelled. Third, an offensive function also exists as the hunted termites were paralyzed. The *C. striatula* workers avoided contact with competing ants or with prey (only slight, fortuitous contacts occurred), showing that the emitted substance acts from a distance. Killing prey from a distance is known in formicine ants that spray formic acid from up to 30 cm away [48]. Also, dolichoderine ants [49], carabid beetles and walkingstick insects can spray their defensive chemical [50], [51], not to speak of spitting cobras that project their venom from a distance to kill their prey [52]. Something similar to the present study was recently published on the ponerine ant *Platythyrea conradti*, but the chemicals are likely secreted by the mandibular gland as the workers drop into a crouch, mandibles wide open [13]. Here, too, these chemicals cause enemies to retreat further and further away and to abandon sugary food sources, while termites, which face the crouching ants, end up by being paralyzed without any contact taking place between the protagonists [13]. Indeed, *Camponotus* ants abandon the sugary substances to avoid their competing *C. striatula* opponents that are equipped with a chemical weapon, while termites are trapped in their defensive behavior and sacrifice themselves [13].

While the *C. striatula* venom acts from a distance both during predation and interspecific competition, in most *Crematogaster* species studied so far the venom is emitted as a froth that accumulates on the spatulate tip of the stinger. In some species, such as *C. scutellaris*, the venom is defensive (not used for prey capture [53] and only used during interspecific confrontations but not intraspecifically as the ants are immune to their own venom [21], [42]). Direct contact with the venom causes enemy ants to retreat, and then rub their antennae and mouthparts against the substrate. Experiments have shown that while contact is not absolutely necessary, the venom does not have an effect at a distance greater than a few millimeters [21].

In the European species *C. scutellaris*, *C. auberti*, and *C. sordidula*, the Dufour gland contents consist of unsaturated keto-acetates. The major compounds derive from an acetylated C21 long chain in *C. scutellaris*, while minor homologs deriving from C19 and C23 chains have also been noted. During their emission, these compounds mix with two enzymes from the poison gland and are transformed into aldehydes. The ants therefore store precursors that have a relatively low level of toxicity, while the final degree of venom toxicity is produced during the simultaneous emission of both the Dufour and the poison gland contents. Also, the acetic acid released during this process acts as an alarm pheromone triggering the recruitment of nestmates at short range [22], [34]. In the present study, we therefore found some similarities in the composition of the *C. striatula* Dufour gland contents such as the C21 chains constituting one of the major compounds; we also noted that nestmates are recruited at short range.

Nevertheless, the originality of our approach resides in the fact that, for the first time, we investigated the Dufour gland contents using ESI-MS, highlighting the presence of a non-volatile compound (1583 Da) which is likely an alkaloid. This chain can split, forming the ion m/z 803, plus another part whose total mass is 781 Da, so an odd value indicating the presence of a nitrogen atom, and corresponding to the supposed alkaloid function. It would be interesting to further investigate this new precursor compound, as well as the processes permitting its transformation into smaller molecules in the final venom composition.

These molecules are likely the source of the relatively strong toxic properties of the *C. striatula* Dufour gland extracts and the final venom toxicity demonstrated in this study during the bioassays and behavioral studies, respectively. Like for *C. scutellaris* [22], the final venom composition is likely more active than the precursor from the Dufour gland. Indeed, in natural conditions, the emitted venom, airborne, paralyzes termites without coming into direct contact with them, while in our bioassays we showed that the Dufour gland contents also had toxic properties when applied topically on the tested termites. It remains true that during the bioassays both the precursor (Dufour gland contents) and the final venom composition triggered the paralysis of the termites relatively quickly, with death following when the contact was prolonged (Table 1).

Several venom alkaloids have been described in several genera belonging, like *Crematogaster*, to the Myrmicinae subfamily [54]–[63]. The hypothesis that alkaloids act at a distance converges with what is known for the genera *Solenopsis*, *Monomorium* and *Megalomyrmex* when workers release volatile venom alkaloids by waving their stingers (i.e., gaster flagging) during interspecific encounters causing their enemies to flee [54]–[56]. Also, volatile compounds have been noted in the poison of some ponerine ants [64], [65]. Nevertheless, the venom is injected during prey capture in all these species whose stingers are sharp [12], [56], [66], [67]. In myrmicine ants with a spatulate stinger (including certain *Crematogaster* species), the venom, applied onto the prey or enemy, acts topically [8], [21], [22], [42], [43], [68]. Formicine and dolichoderine ants are devoid of a stinger. In the Formicinae, venom is sprayed and so acts topically [12]. The hypertrophied pygidial gland of the Dolichoderinae produces iridoid terpenes [62]. *Forelius foetidus* sprays these defensive compounds on its enemies, but the compounds are not visible; their presence was demonstrated by using chemical analyses to record them on the cuticule of attacked competing ants [49]. A similar process likely occurs for the non-volatile parts of *C. striatula* venom (sprayed but not visible), permitting the workers to successfully capture prey. Their ability to repel competitors from a distance may be due to other compounds that are volatile as has been shown experimentally for *C. scutellaris* [21]. In both cases, the fact that the venom acts from a distance permits *C. striatula* workers to avoid contact with all kinds of opponents, and so to easily bypass their defenses.

In conclusion, this study is the first to report that the toxicity of the Dufour gland contents leads to the irreversible paralysis of termites without direct contact occurring between the ants and the prey. These results are promising because they provide a basis from which further studies can be conducted in the search for natural insecticides, including new molecules effective against insects resistant to currently-used insecticides. Indeed, once the paralyzing substance has been successfully identified, a synthetic product can be created that has numerous applications, something that is easier to do with volatile chemicals [69] than with alkaloids.

## References

[1] Davidson DW, Cook SC, Snelling RR, Chua TH (2003). Explaining the abundance of ants in lowland tropical rainforest canopies.. Science.

[2] Hunt J (2003). Cryptic herbivores of the rainforest canopy.. Science.

[3] Blüthgen N, Stork NE, Fiedler K (2004). Bottom-up control and co-occurrence in complex communities: honeydew and nectar determine a rainforest ant mosaic.. Oikos.

[4] Leston D (1973). The ant mosaic, tropical tree crops and the limiting of pests and diseases.. Pest Art News Summ.

[5] Majer JD, LaSalle J, Gauld ID (1993). Comparison of the arboreal ant mosaic in Ghana, Brazil, Papua New Guinea and Australia: its structure and influence of ant diversity.. Hymenoptera and Biodiversity.

[6] Blüthgen N, Stork NE (2007). Ant mosaics in a tropical rainforest in Australia and elsewhere: a critical review.. Aust Ecol.

[7] Dejean A, Corbara B, Orivel J, Leponce M (2007). Rainforest canopy ants: the implications of territoriality and predatory behavior.. Funct Ecosyst Commun.

[8] Richard FJ, Fabre A, Dejean A (2001). Predatory behavior in dominant arboreal ant species: the case of *Crematogaster* sp. (Hymenoptera: Formicidae).. J Insect Behav.

[9] Kenne M, Fénéron R, Djiéto-Lordon C, Malherbe MC, Tindo M (2009). Nesting and foraging habits in the arboreal ant *Atopomyrmex mocquerysi* André (Formicidae, Myrmicinae).. Myrmecol News.

[10] Dejean A, Grangier J, Leroy C, Orivel J (2009). Predation and aggressiveness in host plant protection: a generalization using ants of the genus *Azteca*.. Naturwissenschaften.

[11] Dejean A, Corbara B, Leroy C, Roux O, Céréghino R (2010). Arboreal ants use the “Velcro® principle” to capture very large prey.. PloS ONE.

[12] Hölldobler B, Wilson EO (1990). The ants.

[13] Dejean A (2011). Prey capture behavior in an arboreal African ponerine ant.. PLoS ONE.

[14] Dejean A, Giberneau M (2000). A rainforest ant mosaic: the edge effect (Hymenoptera: Formicidae).. Sociobiology.

[15] Dejean A, Djiéto-Lordon C, Céréghino R, Leponce M (2008). Ontogenic succession and the ant mosaic: an empirical approach using pioneer trees.. Basic Appl Ecol.

[16] Majer JD (1976). The ant mosaic in Ghana cocoa farms: further structural considerations.. J Appl Ecol.

[17] Dejean A, Durand J-L, Bolton B (1996). Ants inhabiting *Cubitermes* termitaries in African rainforests.. Biotropica.

[18] Deblauwe I, Dekoninck W (2007). Diversity and distribution of ground-dwelling ants in a lowland rainforest in southeast Cameroon.. Insect Soc.

[19] Prestwich GD (1984). Defense Mechanisms of Termites.. Ann Rev Entomol.

[20] Buren WF (1958). A review of the species of *Crematogaster*, *sensu stricto*, in North America (Hymenoptera: Formicidae), part I.. J NY Entomol Soc.

[21] Marlier JF, Quinet Y, De Biseau JC (2004). Defensive behaviour and biological activities of the abdominal secretion in the ant *Crematogaster scutellaris* (Hymenoptera: Myrmicinae).. Behav Proc.

[22] Pasteels JM, Daloze D, Boevé JL (1989). Aldehydic contact poisons and alarm pheromone of the ant *Crematogaster scutellaris* (Hymenoptera: Myrmicinae). Enzyme-Mediated Production from acetate precursors.. J Chem Ecol.

[23] Leuthold RH, Schlunegger U (1973). The alarm behaviour from the mandibular gland secretion in the ant *Crematogaster scutellaris*.. Insect Soc.

[24] Maschwitz U, Noirot C, Howse PE, Le Masne G (1975). Old and new trends in the investigation of chemical recruitment in ants.. Pheromones and defensives secretions in social insects.

[25] Kugler C (1978). A comparative study of the Myrmicine sting apparatus (Hymenoptera: Formicidae).. Studia Ent.

[26] Carlet G (1884). Sur le venin des Hyménopteres et ses organes sécréteurs.. C R Acad Sci Paris.

[27] Maschwitz U, Kloft W, Bücherl W, Buckley E (1971). Morphology and function of the venom apparatus of insects – bees, wasps, ants, and caterpillars.. Venomous animals and their venoms, vol. 3.

[28] Hermann HR (1969). The hymenoptera poison apparatus: evolutionary trends in three closely related subfamilies of ants (Hymenoptera: Formicidae).. Georgia Entomol Soc.

[29] Hermann HR, Blum MS (1967). The morphology and histology of the hymenopterous poison apparatus, II: *Pogonomyrmex badius* (Formicidae).. Annals of the Entomological Society of America.

[30] Hermann HR, Blum MS (1967). The morphology and histology of the hymenopterous poison apparatus, III: *Eciton hamatum* (Formicidae).. Annals of the Entomological Society of America.

[31] Hermann HR, Blum MS, Hermann HR (1981). Defensive mechanisms in social Hymenoptera.. Social Insects. 2nd vol, 491p.

[32] Buschinger A, Maschwitz U, Herman HR (1984). Defensive behavior and defensive mechanisms in ants.. Defensive mechanisms in social insects.

[33] Daloze D, Braekman J-C, Vanhecke P, Boevé JL, Pasteels JM (1987). Long chain electrophilic contact poison from the Dufour's gland of the ant *Crematogaster scutellaris* (Hymenoptera, Myrmicinae).. Can J Chem.

[34] Daloze D, Kaisin M, Detrain C, Pasteels JM (1991). Chemical defense in the three European species of *Crematogaster* ants.. Experientia.

[35] Leclercq S, Braekman J-C, Kaisin M, Daloze D, Detrain C (1997). Venom constituents of three species of *Crematogaster* ants from Papua New Guinea.. J Nat Prod.

[36] Leclercq S, de Biseau J-C, Braekman J-C, Daloze D, Quinet Y (2000). Furanocembranoid diterpenes as defensive compounds in the Dufour gland of the ant *Crematogaster brevispinosa rochaï*.. Tetrahedron.

[37] Daloze D, de Biseau J-C, Leclercq S, Braekman J-C, Quinet Y (1998). (13E, 15E, 18Z,20Z)-1-Hydroxypentacosa- 13,15,18,20-tetraen-ll- yn-4-one 1-acetate, from the venom of a Brazilian *Crematogaster* ant.. Tetrahedron Lett.

[38] Leclercq S, de Biseau J-C, Daloze D, Braekman JC, Quinet Y (2000). Five new furanocembrenoids from the venom of the ant *Crematogaster brevispinosa ampla* from Brazil.. Tetrahedron Lett.

[39] Graham JH, Hughie HH, Jones S, Wrinn K, Krzysik AJ (2004). Habitat disturbance and the diversity and abundance of ants (Formicidae) in the Southeastern Fall-Line Sandhills.. J Insect Sci.

[40] Laurent P, Hamdani A, Braekman J-C, Daloze D, Isbell LA (2003). New 1-alk(en)yl-1,3,5-trihydroxycyclohexanes from the Dufour gland of the African ant *Crematogaster nigriceps*.. Tetrahedron Lett.

[41] Laurent P, Braekman J-C, Daloze D (2005). Insect chemical defense.. Top Curr Chemist.

[42] Heredia A, de Biseau J-C, Quinet Y (2005). Toxicity of the venom in three neotropical *Crematogaster* ants (Formicidae: Myrmicinae).. Chemoecology.

[43] Blum MS, Hermann HR, Bettini S (1978). Venoms and venom apparatuses of the Formicidae: Myrmeciinae, Ponerinae, Dorylinae, Pseudomyrmecinae, Myrmicinae, and Formicinae.. Handbuch der experimentellen Pharmakologie. Vol. 48, Arthropod venoms.

[44] Boevé J-L (1994). Injection of venom into an insect prey by the free hunting spider *Cupiennius salei* (Araneae, Ctenidae).. J Zool.

[45] Post hoc analysis for Friedman's Test (R code) (2010). http://www.r-statistics.com/2010/02/post-hoc-analysis-for-friedmans-test-r-code/.

[46] Conover WJ (1980). Practical nonparametric statistics.

[47] R Development Core Team (2011). R: A language and environment for statistical computing.. http://www.R-project.org.

[48] Dumpert K (1981). The Social Biology of Ants.

[49] Scheffrahn R, Gaston LK, Sims JJ, Rust MK (1984). Defensive ecology of *Forelius foetidus* and its chemosystematic relationship to F. ( = *Iridomyrmex*) *pruinosus* (Hymenoptera: Formicidae: Dolichoderinae).. Environ Entomol.

[50] Will KW, Gill AS, Lee H, Attygale AB (2010). Quantification and evidence for mechanically metered release of pygidial secretions in formic acid-producing carabid beetles.. J Insect Sci.

[51] Dossey AT, Gottardo M, Whitaker JM, Roush WR, Edison AS (2009). Alkyldimethylpyrazines in the defensive spray of *Phyllium westwoodii*: a first for order Phasmatodea.. J Chem Ecol.

[52] Berthé AR, de Pury S, Bleckmann H, Westho G (2009). Spitting cobras adjust their venom distribution to target distance.. J Comp Physiol A.

[53] Schatz B, Hossaert-McKey M (2003). Interactions of the ant *Crematogaster scutellaris* with the fig/fig wasp mutualism.. Ecol Entomol.

[54] Jones TH, Blum MS, Fales HM (1982). Ant venom alkaloids from *Solenopsis* and *Monomorium* species. Recent developments.. Tetrahedron.

[55] Obin MS, Vander Meer RK (1985). Gaster flagging by fire ants (*Solenopsis* spp.): functional significance of venom dispersal behavior.. J Chem Ecol.

[56] Adams R (2000). Chemical ecology of *Megalomyrmex* social parasites and their hosts.. http://www1.bio.ku.dk/english/research/oe/cse/personer/rachelle/.

[57] Brand JM, Blum MS, Fales HM, MacConnell JG (1972). Fire ant venoms: comparative analyses of alkaloidal components.. Toxicon.

[58] Jones TH, Devries PJ, Escoubas P (1991). Chemistry of venom alkaloids in the ant *Megalomyrmex foreli* (Myrmicinae) from Costa Rica.. J Chem Ecol.

[59] Andersen AN, Blum MS, Jones TH (1991). Venom alkaloids in *Monomorium “rothsteini”* Forel repel other ants: is this the secret to success by *Monomorium* in Australian ant communities?. Oecologia.

[60] Leclercq S, Braekman JC, Daloze D, Pasteels JM, Vander Meer RK (1996). Biosynthesis of the solenopsins, venom alkaloids of the fire ants.. Naturwissenschaften.

[61] Deslippe RJ, Guo YJ (2000). Venom alkaloids of fire ants in relation to worker size and age.. Toxicon.

[62] Morgan ED (2008). Chemical sorcery for sociality: exocrine secretions of ants (Hymenoptera: Formicidae).. Myrmecol News.

[63] Chen J, Cantrell CL, Shang H-W, Rojas MG (2009). Piperideine alkaloids from the poison gland of the red imported fire ant (Hymenoptera: Formicidae).. J Agric Food Chem.

[64] Morgan ED, Jungnickel H, Keegans SJ, Do Nascimento RR, Billen J (2003). Comparative survey of abdominal gland secretions of the ant subfamily Ponerinae.. J Chem Ecol.

[65] Nikbakhtzadeh MR, Tirgari S, Fakoorziba MR, Alipour H (2009). Two volatiles from the venom gland of the Samsum ant, *Pachycondyla sennaarensis*.. Toxicon.

[66] Greenberg L, Kabashima JN, Allison CJ, Rust MK, Klotz JH (2008). Lethality of red imported fire ant venom to argentine ants and other ant species.. Ann Entomol Soc Amer.

[67] Cerda X, Dejean A, Polidori C (2011). Predation by ants on arthropods and other animals.. Predation in the Hymenoptera: an evolutionary perspective.

[68] Kenne M, Schatz B, Dejean A (2000). Hunting strategy of a generalist ant species proposed as a biological control agent against termites.. Entomol Exp Appl.

[69] Berger RG (2009). Biotechnology of flavours – the next generation.. Biotechnol Lett.

